# Dysbiotic microbes and how to find them: a review of microbiome profiling in prostate cancer

**DOI:** 10.1186/s13046-021-02196-y

**Published:** 2022-01-22

**Authors:** Paul Vinu Salachan, Karina Dalsgaard Sørensen

**Affiliations:** 1 Department of Molecular Medicine, Aarhus University Hospital, 8200 Aarhus N, Denmark; 2 Department of Clinical Medicine, Aarhus University, 8200 Aarhus N, Denmark

**Keywords:** Amplicon sequencing, Metagenome, Metatranscriptome, Microbiome, Prostate cancer

## Abstract

The role of the microbiota in human health and disease is well established, including its effects on several cancer types. However, the role of microbial dysbiosis in prostate cancer development, progression, and response to treatment is less well understood. This knowledge gap could perhaps be implicated in the lack of better risk stratification and prognostic tools that incorporate risk factors such as bacterial infections and inflammatory signatures. With over a decade’s research investigating associations between microbiome and prostate carcinogenesis, we are ever closer to finding the crucial biological link between the two. Yet, definitive answers remain elusive, calling for continued research into this field. In this review, we outline the three frequently used NGS based analysis methodologies that are used for microbiome profiling, thereby serving as a quick guide for future microbiome research. We next provide a detailed overview of the current knowledge of the role of the human microbiome in prostate cancer development, progression, and treatment response. Finally, we describe proposed mechanisms of host-microbe interactions that could lead to prostate cancer development, progression or treatment response.

## Background

The human body comprises of trillions of microorganisms with the estimated bacterial population in the order of 10^13^ cells, resulting in an approximate 1:1 ratio between bacterial and human cells in an average human [1]. It is thus fathomable that the human microbial ecosystem (microbiota) can influence aspects of human health and disease through direct or indirect effects [2], for example by manipulating nutrient uptake and drug metabolism or by inducing systemic inflammatory responses [2–5]. While the resident microbiota is typically associated with beneficial effects to its host, changes to the microbial composition, known as microbial dysbiosis, could be associated with diseases such as inflammatory bowel disease, diabetes mellitus, and obesity (reviewed by [6]). Since inflammation is a signature of many pre-neoplastic and malignant lesions, chronic inflammation has also been implicated in carcinogenesis likely mediated by bacterial toxins as in the case of *Helicobacter pylori* and gastric carcinoma [7]. In fact, a growing body of evidence now suggests a crucial role for microbial dysbiosis in cancer development and progression, including significant associations with both bacterial and viral species [8–12].

Prostate cancer (PCa) is the second most frequent male malignancy worldwide with over 358,000 estimated deaths in 2018 [13]. PCa has a highly heterogeneous clinical course. Most organ confined (localized) PCa have an indolent course with a 5 year overall survival of ~100% even without any treatment. In such cases, active surveillance is recommended. In aggressive forms of PCa where the tumor is still organ confined, complete removal of the prostate through radical prostatectomy and/or radiation therapy is necessary to prevent further spread of the disease. However, the decision whether or not to treat localized PCa is a major clinical challenge as currently there are no accurate tests to distinguish between indolent and aggressive PCa at the localized (early) stages. This leads to an over-treatment of indolent cases and an under-treatment of aggressive PCa, resulting in patient morbidity and mortality. Thus, there is an urgent need for better risk stratification tools, that incorporate other risk factors such as bacterial infections or inflammatory markers [14].

The advent of next generation sequencing (NGS) technologies has opened a new area of PCa research enabling unparalleled access to the genomic and transcriptomic underpinnings in PCa. Utilizing the potential for these technologies, several molecular markers have been proposed to stratify PCa [15], although complete success in this regard is yet to be achieved. A major reason could be the interaction between the neoplastic cells and the tumor microenvironment, which remains dynamic. Prostate microbiota could also be hypothesized to be a major driver enabling differential clinical course in localized PCa. In fact, among the well known risk factors for PCa such as age and ethnicity, factors such as microbial composition has also found a potentially essential place in recent years due to the increased scientific scrutiny of its role in mediating inflammation and thereby driving prostate carcinogenesis and progression [16]. While earlier studies relied on culturing bacteria from the prostate, NGS based methods have enabled genotyping the microbial ecosystem within a prostate for hundreds to thousands of patients in parallel, providing a better overview of the landscape of the PCa associated microbiome.

The aim of this review is twofold, 1) provide the researcher with the necessary technical know-how to perform microbiome analysis, and 2) inform the reader of the advances that have been made in the field of prostate cancer microbiome research. We start out by describing in general how to analyse microbiome data and note several automated pipelines that are available to the researcher. For the sake of simplicity, we restricted our review to the three most frequently used NGS based analysis methodologies (amplicon sequencing, shotgun DNA and total RNA sequencing) that have been widely adopted by the microbiome research community. We next provide a detailed overview of the current knowledge of the role of the human microbiome in PCa development, progression and treatment response that was made possible by some of the aforementioned methodologies. Finally, we describe the proposed mechanisms of host-microbe interactions that could lead to PCa development, progression, or treatment resistance. A clear distinction between microbial association with PCa development (carcinogenesis) and its association with PCa progression (e.g. metastatic dissemination) is difficult to make due to the lack of healthy non-cancer control samples in most studies, and consequently this remains an outstanding question in the field.

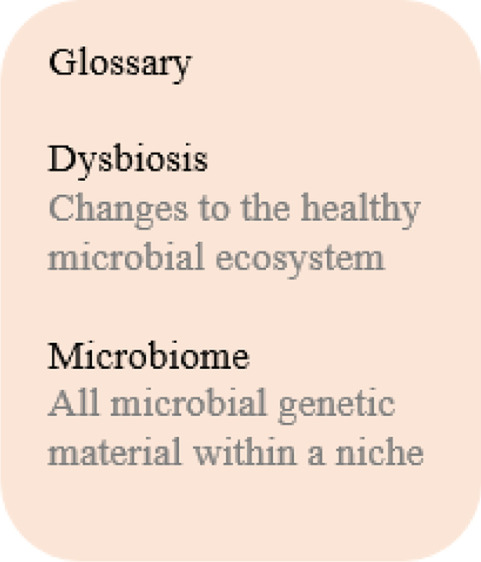



## Methodologies for analyzing the microbiome

**Fig. 1:**
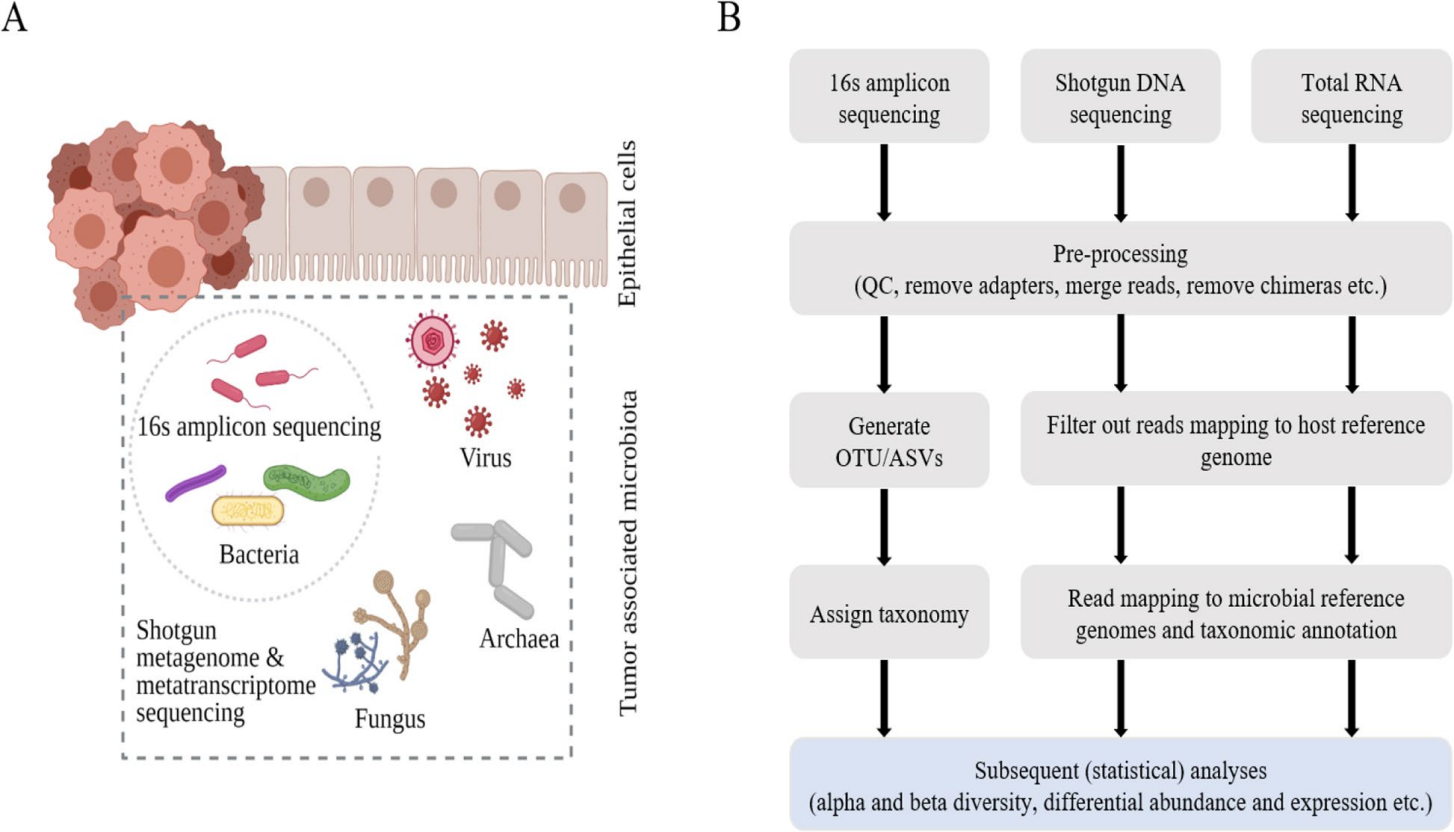
**A**) Prostate tumor microenvironment is shown harboring bacterial, viral, fungal, and archaeal species. 16s amplicon sequencing is useful for profiling e.g. the bacterial taxa, whereas shotgun metagenome (DNA) and metatranscriptome (total RNA) sequencing enables profiling of the entire tumor associated microbiota. **B**) A general workflow for analysing microbiome data outlining the major steps involved for the three main NGS based methods discussed in this review. QC, quality control. OTU, operational taxonomic unit. ASV, amplicon sequence variants. Figure 1A is inspired by [19]. Image created partly using Biorender.com

Most of the current research enumerating the microbial species present in the prostate and various other body sites utilize NGS based methodologies as opposed to the culture based techniques employed during the last century, which could detect only species that could be cultured. Three main methodologies are most commonly used now (Fig. 1A). These include amplicon sequencing, shotgun DNA sequencing, and RNA sequencing based methodologies. An in depth explanation of the analysis methodologies and best practises for microbiome research is beyond the scope of this review, but we direct readers to other published reviews [17, 18]. A graphical summary of the general steps involved in microbiome sequence analysis is shown in Fig. 1B.

### Amplicon sequencing

Perhaps the most straight forward method to profile the prokaryotic taxa is to sequence the DNA encoding for the 16s rRNA gene, which is highly conserved among all prokaryotes. Typically, one or more variable regions (V1-V9) of the 16s DNA is amplified and sequenced. Next, for multiple samples sequenced in parallel, the raw sequencing reads are de-multiplexed (e.g. using sabre: https://github.com/najoshi/sabre) and quality checked (e.g. using FastQC [20]) to remove low quality sequences and adapter/primer sequences (e.g. using Trimmomatic [21]). If using paired-end sequencing data, the read pairs are merged, chimeras removed and either an operational taxonomic unit (OTU) or an amplicon sequence variant (ASV) table is generated, which records the number of times each OTU/ASV was observed. While OTUs have been traditionally used as a unit for clustering similar sequences, finer resolution can be achieved using ASVs, that are accurate down to the level of single-nucleotide differences [22]. Finally, taxonomy is assigned to the OTU/ASVs using reference microbial databases such as the SILVA 16s rRNA gene database [23]. These steps are typically performed within dedicated pipelines, such as DADA2 [24], mothur [25], or qiime2 [26]. The data is now ready for further exploratory analyses as well as statistical testing of species compositional differences.

Phyloseq [27] is a powerful R [28] package that is widely used for microbiome analysis of taxonomy-assigned OTU/ASV count data. Here it is possible to analyze the alpha diversity (e.g. total number of species and their relative proportions within a patient or sample group) and beta diversity (compositional difference between populations) using R packages such as vegan [29]. Additionally, differential abundance can be estimated using R packages such as DESeq2 [30] or a Conda formula such as LEfSe [31].

A limitation to amplicon sequencing is that only a particular gene region is amplified, which can bias diversity estimates depending on which variable region is selected. Further, different organisms can have different 16s gene copy numbers [32] which can bias microbial abundance estimates even though tools such as PICRUSt [33] have been developed in an attempt to correct for such biases [34].

### Shotgun DNA and RNA sequencing

Using whole genome sequencing or total RNA sequencing as a way to detect non-host DNA/RNA has become a popular alternative to amplicon sequencing, as it enables species-level identification of organisms and generates a complete genome as well as a transcriptome for all the species, meaning that we can obtain information regarding the functional significance of the microbiome. Further, bacterial, viral, fungal and other archaeal reads can all be obtained using these methodologies, making them ideal for microbiome analyses. RNA sequencing based metatranscriptomic analysis can also shed light into which species are contributing actively to the expression profile of the tissue, whereas DNA sequencing based metagenome analysis captures all species, even though they might not be actively contributing to the tissue phenotype. Often, a combination of these two methodologies is required to understand the underlying tumor biology in the context of microbiome interactions with the tumor microenvironment.

The bioinformatics workflow for metagenome or metatranscriptome analyses are similar to that for amplicon sequence based analyses, however with some key differences. Assuming that one has sequenced the entire microbial DNA/RNA pool from e.g. a human tissue sample, to an acceptable coverage, and has performed the pre-processing steps that are generic to raw sequence read analysis, including ribosomal RNA removal and paired-end read merging, then the first step is to align the reads to the reference human genome, e.g. hg38 [35] using tools such as bwa [36]. Reads that do not map to the reference could be considered to be of non-host origin. These reads can then be mapped to reference genomes in the bacterial, viral, fungal, and archaeal sequence databases (e.g. SILVA [23], NCBI RefSeq [37]) and annotated with tools such as DIAMOND [38]. Finally the annotated reads are aggregated to generate the read/taxa count tables which can subsequently be analysed similar to the amplicon sequence generated OTU/ASV count tables.

Many automated bioinformatics pipelines such as Sunbeam [39], MetaWRAP [40] and SqueezeMETA [41] are available for metagenomic analysis, whereas workflows such as IMP [42], SAMSA2 [43], and MetaTrans [44] have been made for analysing the metatranscriptome. An overview of some of these pipelines, including their capabilities and shortcomings have been reviewed by others previously [45]. The steps described above are generic, and variations to these methodologies do exist but are beyond the scope of the current review.

Using these aforementioned methodologies, several studies have investigated the PCa associated microbiome as described in the following sections.

## Prostate cancer and the human microbiome

**Fig. 2:**
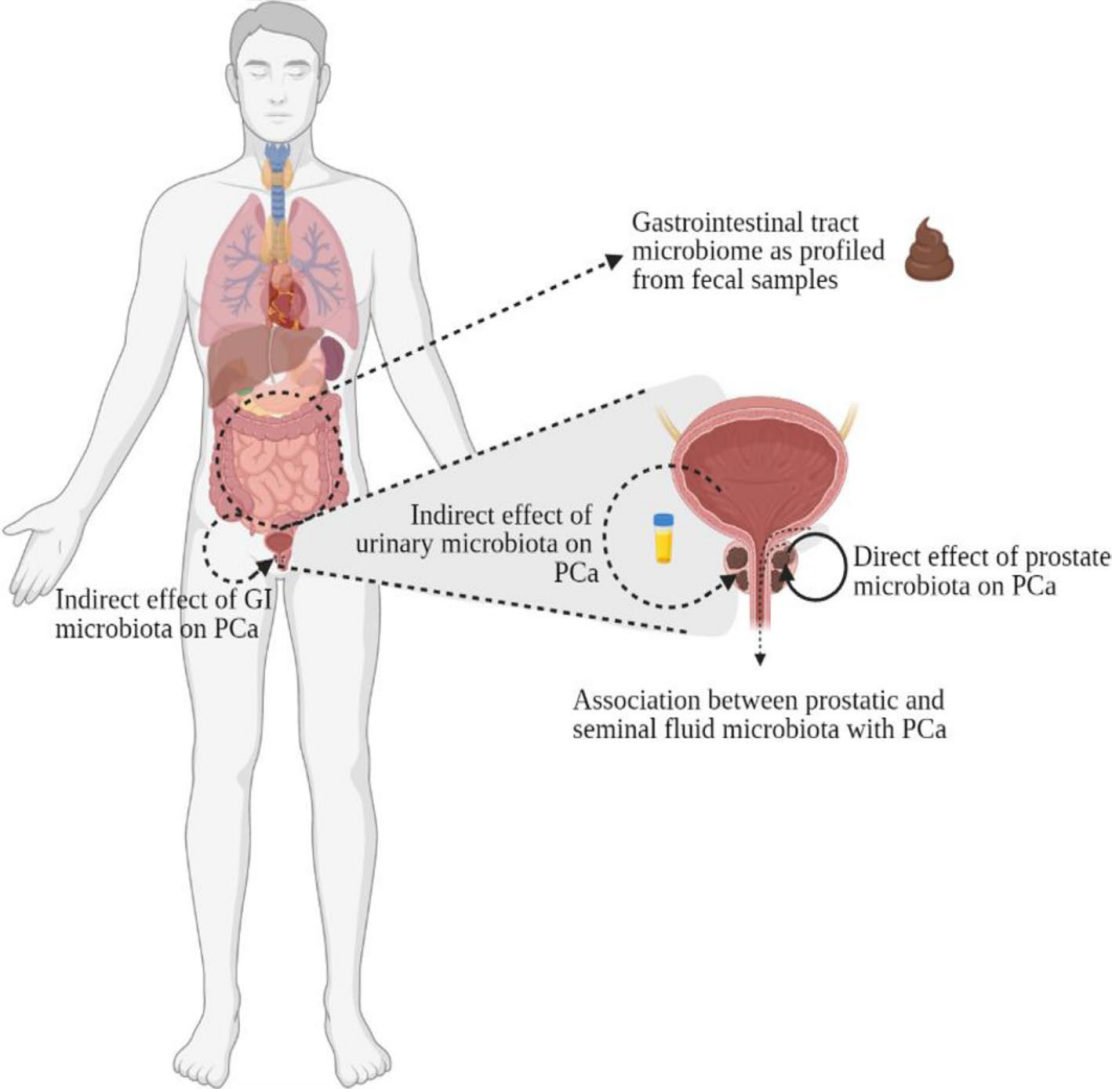
Human microbiome from different body sites have been investigated for its association with prostate cancer (PCa). GI, gastrointestinal. Image created using Biorender.com

Since 2015 there has been a steady rise in the number of publications looking at the association between the human microbiome and prostate cancer development, progression, and treatment outcome. While most research has focussed on the so called direct effect on PCa of the microbiome in the prostate tissue, others have also investigated associations between PCa and the core microbiota from different body sites, the so called indirect effects [2], as depicted in Fig. 2. These have mainly focussed on the effect of the gastrointestinal microbiota and the urinary microbiome on neoplastic transformation of prostatic epithelia [46, 47], but also include studies evaluating associations between PCa and prostatic and seminal fluid microbiomes [48, 49].

Table 1 provides a list of NGS based studies since 2015 investigating microbial dysbiosis associated with PCa. These studies were selected based on a PubMed search for ‘prostate cancer microbiome’ resulting in 159 records since 2015 (as of July 2021). Of these, review articles (n=58) were excluded, and original studies (n=20) profiling the prostate tissue, gut, urinary, seminal fluid and prostatic fluid microbiomes in relation to prostate cancer were selected. A recent study from our group is also discussed in this context.

**Table 1:** Selected publications since 2015 investigating microbial dysbiosis and associations with PCa

Study	Design	Tissue	Sample size	Methodology	Main findings	Significance test	Shortcomings
***Prostate microbiome***							
Salachan et al., 2022; in press	Comparison of microbiome between benign (AN) and malignant tumor tissue samples from 94 RP patients	Fresh frozen tissue	83 malignant and 23 adjacent benign (n=106)	Metatranscriptomic analysis of total RNA sequencing data	Significantly increased abundances of *Shewanella* and decreased abundances of *Bacteroides fragilis*, *Saimiriine betaherpesvirus*, *Staphylococcus saprophyticus*, *Vibrio parahaemolyticus* in malignant as compared to benign tissue samples.	Wald test within DESeq2, *P* < 0.01, LFC > |0.58|	Lack of true normal comparison.
Ma et al., 2020 [50]	Comparison of microbiome between benign (AN) and malignant tumor tissue samples from RP patients	Fresh frozen tissue	242 malignant and 52 adjacent benign (n=294)	Whole-transcriptome RNA sequencing	*Listeria monocytogenes, Methylobacterium radiotolerans JCM 2831, Xanthomonas Listeria monocytogenes, Methylobacterium radiotolerans JCM 2831, Xanthomonas albilineans GPE PC73*, and *Bradyrhizobium japonicum* are overrepresented in the tumor tissue as compared to the benign tissue samples.	Kruskal-Wallis test, *P* < 0.05	Lack of true normal comparison.
Feng, Ramnarine et al., 2019 [53]	Comparison of microbiome between benign (AN) and malignant tumor tissue samples from 65 RP patients	Fresh frozen tissue	65 malignant and 65 adjacent benign (n=130)	Metagenomic and metatranscriptomic analyses	*Escherichia*, *Propionibacterium*, *Acinetobacter*, and *Pseudomonas* were most abundant in the prostate. No species found to be differentially abundant, and no difference in alpha or beta diversity could be found.	Wilcoxon signed rank test, *P* < 0.05, FC > 2	Lack of negative control. *Propionibacterium* is a known sequencing contaminant.
Banerjee et al., 2019 [56]	Comparison of microbiome between prostate adenocarcinoma and BPH tissue samples from 50 RP and 15 TURP (BPH) patients	Formalin-fixed paraffin-embedded	50 malignant and 15 BPH (n=65)	Array-based metagenomic and capture sequencing	Malignant samples were significantly associated with the bacterial phyla such as Proteobacteria, Firmicutes, Actinobacteria, and Bacteroidetes, fungal phyla such as Ascomycota, and Zygomycota, parasitic phyla such as Nematoda, and Sarcomastogophora, and group I and group IV viruses.	t-test, *P* < 0.05, LFC > 1	Lack of true normal comparison.
Feng, Jaratlerdsiri et al., 2019 [54]	Comparison of prostate tissue microbiome between African and Australian samples from PCa patients	Fresh frozen tissue	6 African and 16 Australian malignant samples (n=22)	Metagenomic analysis	Most abundant genera in prostate belong to *Escherichia, Propionibacterium,* and *Pseudomonas.* African samples had significantly increased bacterial richness as compared to Australian samples.	t-test, *P* < 0.01	Small sample size. *Propionibacterium* is a known sequencing contaminant.
Miyake et al., 2019 [76]	Comparison of specific microbial taxa between prostate adenocarcinoma and BPH tissue samples from 45 RP and 33 TURP (BPH) patients	Formalin-fixed paraffin-embedded	45 malignant and 33 BPH (n=78)	PCR screening	Increased rates of *Mycoplasma genitalium* was associated with Pca.	Mann-Whitney U test, *P* < 0.05	Limited number of species tested.
Cavarretta et al., 2017 [77]	Comparison of microbiome between tumoral, peri-tumoral, and non-tumoral tissue samples from 16 RP patients	Formalin-fixed paraffin-embedded	16 tumoral, 16 peri-tumoral and 16 non-tumoral (n=48)	Ultradeep pyrosequencing	Actinobacteria, Firmicutes and Proteobacteria are the most abundant taxa in the prostate. Significantly increased abundances of *Staphylococcus* and decreased abundances of *Streptococcus* in tumoral + peri-tumoral tissue as compared to non-tumoral tissue samples.	Wilcoxon rank-sum test, *P* < 0.05	Lack of negative control.
Yow et al., 2017 [65]	Comparison of microbiome between benign (AN) and malignant tumor tissue samples from 10 RP patients	Fresh frozen tissue	10 malignant and 10 benign (n=20)	16s rRNA amplicon sequencing and total RNA sequencing	*Enterobacteriaceae, Escherichia* and *Propionibacterium* acnes identified as most common in both malignant and benign samples. Endogenous retroviruses could be detected in both malignant and benign samples.	n/a	*Propionibacterium* is a known sequencing contaminant.
Chen and Wei, 2015 [64]	Comparison of 7 viral and 1 bacterial species between tumoral and benign samples from 20 Western RP and 14 Chinese patients	n/a	20 malignant and 10 matched AN from Western patients, 14 malignant and 14 matched AN tissue from Chinese patients	RNA sequencing	*Propionibacterim acnes* genes detected in both tumor and benign tissue. No virus detected in Western patients but few viruses detected in Chinese samples.	n/a	Limited number of species tested. No information whether fresh-frozen or FFPE tissue used.
***Gastrointestinal Microbiome (Fecal)***							
Matsushita et al., 2021 [78]	Comparison of microbiome between high and low risk PCa group	Frozen fecal samples from a rectal swab	96 patients with PCa and 56 patients without PCa (n=152)	16s rRNA amplicon sequencing	Increased relative abundance of *Rikenellaceae, Alistipes*, and *Lachnospira* in high compared to low risk group.	Mann-Whitney U test or chi-squared test, *P* < 0.05	No sequencing controls.
Li et al., 2021 [79]	Comparison of microbiome between patients who underwent RP and those undergoing ADT	Frozen fecal samples	56 patients on ADT and 30 patients who underwent RP (n= 86)	16s rRNA amplicon sequencing	Increased relative abundance of *Ruminococcus gnavus* and *Bacteroides* spp. and decreased abundance of *Lachnospira* and *Roseburia* in patient undergoing ADT.	Kruskal-Wallis test, Wilcoxon rank-sum test or LDA within LEfSe, *P* < 0.05	Lack of independent validation.
Daisley et al., 2020 [62]	Comparison of microbiome between patients not receiving any active treatment, those receiving ADT alone and those receiving both ADT and orally administered AA	Frozen fecal samples	33, 21, and 14 samples from patients not receiving any active treatment, those receiving ADT alone and those receiving both ADT and orally administered AA, respectively (n=68)	16s rRNA amplicon sequencing	Decreased relative abundance in *Corynebacterium* and increased relative abundance of *Akkermansia muciniphila* in patients undergoing ADT+AA compared to controls.	Wilcoxon rank-sum test, *P* < 0.05	Only bacterial species profiled.
Liu and Jiang, 2020 [63]	Comparison of microbiome between paired samples collected before ADT (HSPC) and after ADT on progression to CRPC	Frozen fecal samples	21 samples before ADT (HSPC) and 21 samples after ADT at CRPC (n=42)	16s rRNA amplicon sequencing	Increased abundance of *Phascolarctobacterium* and *Ruminococcus* in samples collected after ADT from patients who progressed to CRPC as compared to the samples before ADT.	LDA within LEfSe, log_10_ LDA score > 2	Small sample size.
Alanee et al., 2019 [60]	Comparison of microbiome between patients with benign and malignant disease identified through trans-rectal biopsy of the prostate	Frozen fecal samples	16 patients with benign and 14 patients with PCa (n=30)	16s rRNA amplicon sequencing	No clustering of samples based on benign and malignant biopsy. Higher abundance of *Bacteroides* in patients with PCa compared to controls.	Kruskal-Wallis test, *P* < 0.05	Small sample size. Negative controls not sequenced.
Sfanos et al., 2018 [61]	Comparison of microbiome from control, benign, localized PCa, biochemically recurrent PCa, and metastatic PCa patients	Frozen fecal samples	6 control, 3 benign, 7 localized PCa, 7 biochemically recurrent PCa, and 7 metastatic PCa patients (n=30)	16s rDNA amplicon sequencing	Increased abundance of *Akkermansia muciniphila* and *Ruminococcaceae* spp. in men taking ATT compared to those who did not.	Negative binomial test within DESeq, *P* < 0.05	Small sample size.
Liss et al., 2018 [47]	Comparison of microbiome between patients with and without PCa identified through trans-rectal biopsy of the prostate	Rectal swab kept frozen in PBS	64 samples from patients with PCa and 41 samples from patients without PCa (n=105)	16s rRNA amplicon sequencing	*Bacteroides* and *Streptococcus* species were enriched in samples from patients with PCa compared to patients without.	t-test, *P* < 0.05	Use of rectal swabs instead of stool collection limits DNA yield.
Golombos et al., 2018 [59]	Comparison of microbiome between patients with benign prostatic conditions (controls) and clinically localized prostate cancer	Frozen fecal samples	8 men with benign and 12 men with PCa (n=20)	Metagenomics analysis	Higher relative abundance of *Bacteriodes massiliensis* observed in men with PCa compared to controls. Higher relative abundance of *Faecalibacterium prausnitzii* and *Eubacterium rectalie* among controls.	Kruskal-Wallis test, Wilcoxon rank-sum test or LDA within LEfSe, *P* < 0.05, log_10_ LDA score > 2	Small sample size.
***Urinary microbiome***							
Shreshtha et al., 2018 [46]	Comparison of microbiome between patients with positive vs. negative biopsies for Pca	Urine processed within 4 hours of collection	61 samples from men with PCa, 63 from men without PCa, and 5 from men who had negative first and positive second biopsy (n=129)	16s rDNA sequencing	*Propionibacterium lymphophilum* identified to have significantly higher abundance in cancer vs. benign samples.	Fisher exact test, *P* < 0.05	Lack of true normal urine samples.
Alanee et al., 2019 [60]	Comparison of microbiome between patients with benign and malignant disease identified through trans-rectal biopsy of the prostate	Frozen first voided urine samples after prostatic massage	16 patients with benign and 14 patients with PCa (n=30)	16s rRNA amplicon sequencing	Higher abundance of *clostridium XVIII* & *IV*, *lachnospira*, *acetanaerobacterium*, and *faecalibacterium* in the patients with PCa compared to controls.	Kruskal-Wallis test, *P* < 0.05	Small sample size. Negative controls not sequenced.
Yu et al., 2015 [49]	Comparison of microbiome between patients with BPH and PCa	Frozen urine	21 samples from patients with BPH and 13 samples from patients with PCa (n=34)	16s rDNA and PCR-DGGE and qPCR	*E. coli* and *Enterococcus* are present in significantly lower number in the urine of men with PCa compared to BPH.	ANOVA or t-test, *P* < 0.05	Small sample size.
***Prostate and seminal fluid microbiome***							
Ma et al., 2019 [48]	Comparison of microbiome between patients with PCa and those without	Fresh frozen prostatic fluid	32 samples from PCa and 27 samples from non-PCa men (n=59)	16s rRNA amplicon sequencing	Reduced microbial diversity in PCa samples. Increased proportions of L*actococcus, Carnobacterium, Streptococcus, Geobacillus*, and *Enterobacter,* and decreased proportions of *Cronobacter, Alkaliphilus*, and *Paenibacillus* in samples from patients with PCa compared to those without.	Friedman’s test or Wilcoxon rank-sum test, *P* < 0.05	Difficult to control bacterial contamination from urinary tract.
Chen and Wei, 2015 [64]	Comparison of 7 viral and 1 bacterial species between biopsy proven and biopsy negative samples from 12 individuals	Non-sperm fraction of seminal fluid freshly collected	1 pooled sample each from 6 biopsy proven and 6 biopsy negative men	Small RNA sequencing	*Propionibacterium acnes* genes detected in biopsy proven but not in biopsy negative pooled sample.	n/a	Limited number of species tested.
Yu et al., 2015 [49]	Comparison of microbiome between patients with BPH and PCa	Frozen expressed prostatic secretions and seminal fluid	Pooled sample from patients with BPH (n=21) or PCa (n=13)	16s rDNA and PCR-DGGE and qPCR	EPS of PCa patients were rich in *Bacteroidetes* bacteria, *Alphaproteobacteria, Firmicutes* bacteria, *Lachnospiraceae, Propionicimonas, Sphingomonas,* and *Ochrobactrum. E. coli* was present in significantly large number in the EPS and seminal fluid, whereas *Enterococcus* was present in significantly higher number in the seminal fluid of men with PCa compared to BPH.	ANOVA or t-test, *P* < 0.05	Small sample size.

AN, adjacent normal. RP, radical prostatectomy. BPH, benign prostatic hyperplasia. TURP, trans-urethral resection of the prostate. qPCR, quantitative real-time polymerase chain reaction. PCR-DGGE, polymerase chain reaction-denaturing gradient gel electrophoresis. LDA, linear discriminant analysis. LEfSe, LDA effect size. LFC, log_2_ fold change. n/a, not available.

Note1: Some of the genera identified above have been detected in the prostate in most studies. These include *Escherichia*, *Propionibacterium*, *Acinetobacter*, and *Pseudomonas.* However, these are also reported to be common contaminants in multiple sequencing-based microbiome studies [80].

Note 2: *P* value thresholds corrected for multiple testing are reported when possible.

### Prostate microbiome and PCa

With over a decade of research into understanding the role played by the prostatic microbiota in PCa pathophysiology, we are yet to find any causative organisms directly linked to prostate carcinogenesis, despite several studies indicating an association between certain species and the risk of PCa.

We recently investigated the association between the prostate microbiome and PCa using a metatranscriptomic approach based on total RNA sequencing data from 94 PCa patients who underwent curatively intended radical prostatectomy for localized PCa (Salachan et al., 2022; in press). In order to investigate potential dysbiosis associated with PCa, we systematically compared the microbiomes between benign (adjacent normal (AN)) and malignant prostate tissue samples, between less vs. more-aggressive PCa, and between patients who suffered a biochemical disease recurrence compared to those who did not.

We revealed considerable dysbiosis associated with PCa. Notably, species such as *Bacteroides fragilis*, *Saimiriine betaherpesvirus*, *Staphylococcus saprophyticus*, and *Vibrio parahaemolyticus* had a significantly reduced abundance in the malignant as compared to the benign prostate tissue samples. Similarly, we observed a significant increase in the abundance of *Shewanella* in the malignant as compared to the benign prostate tissue samples, suggesting a perhaps important biological link between the prostate microbiota and PCa development. Within malignant tissue samples, those that had a higher abundance of *Shewanella* were associated with dysregulated host immune response, likely mediated by a decrease in enrichment of dendritic cells. We also observed a significant increase in the abundance of *Microbacterium* species in the T3 tumor samples as compared to the T2 samples, suggesting an association between advanced pathological stage and dysbiosis. While, the lack of true normal comparisons is a limitation to this study, obtaining tissue from healthy individuals is hard and ethically challenging.

Other NGS-based studies have also correlated the abundance of microbial species with known risk factors for PCa. Using large scale whole transcriptome RNA sequencing data obtained from The Cancer Genome Atlas (TCGA) for 242 prostate adenocarcinoma (PRAD) patients from the United States and Germany, a recent study [50] found specific microbes such as *Listeria monocytogenes*, *Methylobacterium radiotolerans JCM 2831*, and *Xanthomonas albilineans GPE PC73* to be negatively correlated with Gleason score, Tumor-Node-Metastasis (TNM) stage, and prostate-specific antigen (PSA) level, respectively. Microbes such as *L. monocytogenes* are known to play anti-tumor roles in PCa, for example by stimulating the innate and adaptive immune response [51]. The authors suggest that an over-representation of *L. monocytogenes* in the tumor as compared to AN samples, indicates a strategy wherein the microbes outcompete the tumor cells in the tumor microenvironment, enable recruitment of immune cells and thereby mitigate tumor growth.

Furthermore, in this study [50], *Nevskia ramose* was found to have a positive correlation with Gleason score although its significance in PCa is not understood. *Staphylococcus aureus* was also found to be positively correlated with genomic alterations including amplifications in chromosome 19 and deletions in chromosome 15 and was associated with dysregulated immune-associated genes in this study [50], indicating its pro-tumor roles by inducing inflammatory responses. Strikingly, 234 microbes were significantly associated with elevated levels of PSA [52], the highest number reported from any of the studies included in this review. Such an extent of microbial dysbiosis adds to our knowledge of the crucial role played by microbes in maintaining homeostasis. The lack of true normal comparison is a major limitation to this study [50], preventing us from understanding whether the AN samples truly reflect the normal prostate tissue from men without PCa.

Using both a metagenomic and a metatranscriptomic approach, a study investigating microbial dysbiosis in a Chinese cohort of 65 PCa patients [53] could not differentiate the microbiomes between matched tumor (n=65) and benign (AN) samples (n=65) or between low (n=29) and high (n=36) Gleason score samples. However, both the metagenome and metatranscriptome identified a set of abundant species comprising the core microbiome of the prostate. These included *Escherichia*, *Propionibacterium*, *Acinetobacter* and *Pseudomonas*. Further investigation of their expression profiles revealed strong correlation between ten *Pseudomonas* genes and eight host small RNA genes. The authors noted that three of the host small RNA genes may be negatively associated with metastasis as they observed a high expression of these genes in a subset of patients with low rates of metastasis [53]. However, this was not validated in a larger independent cohort.

In another metagenomic study comparing PCa microbiome of patients from different geographic/ethnic origins [54], significant increase in the richness of bacterial species was observed for prostate tumor samples from African men (n=6) when compared to samples from Australian men (n=16), with the former enriched for genera such as *Escherichia* and *Acidovorax*. This is perhaps not surprising considering the presence of geographic and ethnic variation in the composition of the human microbiome [55]. The small number of samples is a major challenge to this study [54].

A major stride towards establishing a microbiome signature for PCa was made using an array-based metagenomics and capture sequencing method [56]. The study identified microbial signatures from bacteria, viruses, fungi, and parasites within formalin fixed paraffin embedded prostate tissue samples from American men with PCa. Compared to the benign prostatic hyperplasia control samples (n=15), the malignant samples (n=50) were significantly associated with bacterial phyla such as *Proteobacteria*, *Firmicutes*, *Actinobacteria*, and *Bacteroidetes*, fungal phyla such as *Ascomycota*, and *Zygomycota*, parasitic phyla such as *Nematoda*, and *Sarcomastogophora*, and group I and group IV viruses [56]. Using hierarchical clustering, the authors identified three distinct PCa-specific microbiome signatures that were correlated with disease aggressiveness, suggesting diagnostic and prognostic potential for these signatures [56].

### Gastrointestinal microbiome and PCa

Of the indirect effects of the microbiome on prostate carcinogenesis, the association between gut microbiota and PCa has been studied the most. The human gastrointestinal tract harbors the majority of the bacterial population in humans surpassing that of any other bodily site by several orders of magnitude [1]. Coevolution of the gut microbiota enabled symbiotic relationships to exist between humans and the gut microbes, wherein the host provides a conducive environment within the intestine for microbial growth and in turn the microbes aid in digestion [57]. Dysbiosis of the gut microbiota has been implicated in various gastric carcinomas such as colorectal cancer [58], whereas its indirect effect on PCa is less well understood.

In a prospective case-control metagenomic study from 2018, stool samples from 20 Caucasian men with either benign prostatic conditions (n=8) or localized PCa (n=12) were analysed to evaluate their gut microbiome [59]. The study identified biologically significant differences in the composition of the gut microbes between men with PCa compared to men with other benign conditions in the prostate. This included a higher relative abundance of *Bacteriodes massiliensis* as well as decreased relative abundances of *Faecalibacterium prausnitzii* and *Eubacterium rectalie* in the stool from men with PCa compared to the controls. A major limitation to this study [59] is the small sample size and the lack of validation in an independent cohort.

Similarly, enrichment of *Bacteroides* and *Streptococcus* species in rectal swabs from patients with PCa (n=64) compared to non-cancer controls (n=41) were found in another study utilizing 16s rRNA amplicon sequencing [47]. The cohort consisted of a mix of races including White, African-American and Latino. This study further generated a microbiome score based on the microbial metabolic profiles, which held predictive potential for PCa risk, indicating the usefulness of the fecal microbial data as a minimally invasive diagnostic tool. However, a recent study [60] utilizing 16s rRNA amplicon sequencing was unable to separate fecal samples obtained from White, non-Hispanic men with benign (n=16) vs. malignant biopsy (n=14) based on their microbial profiles. Nevertheless, a higher abundance of *Bacteroides* species was observed in patients with PCa compared to control men without PCa in this latter study [60], corroborating the findings from the other studies [47, 59].

While many studies have focused on the role of microbes in PCa development and progression, few have also investigated the changes to the microbiota following treatment against PCa. Using 16s rDNA amplicon sequencing, one such study [61] evaluated the fecal microbiota from 30 PCa patients undergoing gonadotropin releasing hormone agonist/antagonist (GNRH, n=5) or androgen receptor axis-targeted therapy (ATT, n=9) or no treatment (n=16). The study reported altered GI microbiota in men undergoing oral ATT, which could perhaps influence the clinical response to ATT. Specifically, *Akkermansia muciniphila* and *Ruminococcaceae* spp. were over-abundant in the fecal specimens of patients undergoing oral ATT compared to the other groups [61].

Similar findings were also observed in two other recent studies employing 16s rRNA amplicon sequencing methodology [62, 63]. Using a Canadian cohort of 68 castration resistant PCa (CRPC) patients undergoing androgen deprivation therapy (ADT) alone (n=21), ADT along with oral abiraterone acetate (AA, n=14) or no treatment controls (n=33), the first study demonstrated depletion of *Corynebacterium* spp. in patients undergoing ADT, and an enrichment of *Akkermansia muciniphila* in patients taking oral AA, both compared to patients not receiving any form of treatment [62]. The second study [63] compared the fecal microbiome before (n=21) and after ADT (n=21) in a castration resistant setting using a Chinese cohort of 21 CRPC patients. The authors observed a significant increase in the abundance of *Phascolarctobacterium* and *Ruminococcus* as well as for several other bacterial species in patients who received ADT. Whether these insights could be exploited to enhance patient response to ADT needs to be investigated further.

### Urinary microbiome and PCa

Few studies have investigated the association between the urinary microbiota and PCa. Urine samples are readily available, non-invasive, and have a higher patient compliance. Associations between the urinary microbiota and PCa can potentially serve as a biomarker that can be incorporated into pre-biopsy models to better predict PCa risk [60].

One of the earliest studies evaluating the urinary microbiome and PCa using 16s rDNA PCR-denaturing gradient gel electrophoresis (DGGE) found significantly decreased abundance of *E. coli* and *Enterococcus* in the urine from PCa patients (n=13) compared to men (n=21) with benign prostatic hyperplasia (BPH) [49] in a Chinese cohort of men. In another recent study of 30 White, non-Hispanic men [60], species such as *Clostridium XVIII* & *IV*, *lachnospira*, *Acetanaerobacterium*, and *Faecalibacterium* were found to be in significantly higher abundance in the urine from patients with PCa (n=14) compared to benign controls (n=16) as identified using 16s rDNA amplicon sequencing [60]. A prior study analysed urine samples from 129 American men using 16s rDNA sequencing and identified *Propionibacterium lymphophilum* to have significantly higher abundance in PCa patients (n=61) vs. control men (n=63) who did not have PCa [46]. However, a major limitation to all these studies [46, 49, 60] is the lack of independent validation and the lack of consensus between studies. More streamlined research in this field is required before definitive conclusions can be made about any association (or lack thereof) between the urinary microbiota and PCa.

### Prostatic and seminal fluid microbiome and PCa

While it is interesting to profile the prostatic and seminal fluid microbiomes for associations with PCa due to their close proximity to the prostate gland, controlling for contamination from the urinary tract in these samples is often difficult. Few studies have evaluated the prostatic and seminal fluid microbiomes in PCa [48, 49, 64]. Using PCR-DGGE, one study [49] found a significantly increased abundance of *Bacteroidetes*, *Alphaproteobacteria*, *Firmicutes*, *Lachnospiraceae*, *Propionicimonas*, *Sphingomonas*, and *Ochrobactrum*, and a decreased abundance of *Eubacterium* and *Defluviicoccus* in the expressed prostatic secretions (EPS) from Chinese PCa patients (n=13) compared to Chinese men with BPH (n=21). In this study, qPCR detection of *E.coli* and *Enterococcus* revealed that *E. coli* was present in significantly higher number in the EPS and seminal fluid, whereas *Enterococcus* was present in significantly higher number in the seminal fluid of men with PCa compared to men with BPH [49].

Using 16s rDNA amplicon sequencing, another study found increased proportions of *Lactococcus*, *Carnobacterium*, *Streptococcus*, *Geobacillus*, and *Enterobacter*, and decreased proportions of *Cronobacter*, *Alkaliphilus*, and *Paenibacillus* in prostatic fluid samples from patients with PCa (n=32) compared to men without PCa (n=27) in a Chinese cohort [48], whereas *Propionibacterium acnes* genes were detected in the non-sperm fraction of the seminal fluid from Australian men with PCa (pooled sample from 6 men) but not in men without PCa (pooled sample from 6 men) using small RNA sequencing in a different study [64]. Most of these studies [49, 64] are limited by the small sample sizes and the use of pooled samples. Nevertheless, some interesting observations were made, necessitating further research into this area.

## Functional role of specific microbes in PCa

A number of studies have investigated the possible functional role of specific microbes in relation to PCa due to their inflammatory potential and frequent detection in prostatic tissue (Table 2). *Cutibacterium acnes* (formerly, *Propionibacterium acnes*) is a skin-associated commensal that has been detected in the prostate of men with PCa in several studies [53, 65] with few studies reporting a higher prevalence in patients with PCa compared to men without [66, 67]. *C. acnes* has also been associated with chronic inflammation in the prostate of men with PCa [68] and shown to induce acute and chronic inflammation in mice inoculated with human prostatectomy-derived *C. acnes* isolates [69]. Evidence from cell-based experiments suggests that *C. acnes* can induce cell proliferation [66] and the secretion of cytokines and chemokines such as IL-6 and IL-8 [70, 71], which are crucial for maintaining active inflammation. However, a later study failed to observe any statistical difference in IL-6 secretion between men with vs. without *C. acnes* infection [72]. In another study, prostatic epithelial cell lines infected with *C. acnes* responded via activation of transcription factors such as NF-κB and STAT3 [70], which are associated with cellular proliferation and tumor growth in various cancers, such as PCa and colon cancer [73, 74]. Others have also provided evidence for *C. acnes*-induced production of reactive oxygen species by keratinocytes in the skin, thereby inducing oxidative stress response and inflammation [75], an event that could perhaps be replicated in the prostate leading to pre-cancerous transformation of the prostatic epithelia [70]. Taken together, all of this evidence points to a possible role for *C.acnes*-induced inflammation in PCa development or progression.

**Table 2:** Functional roles of selected microbes in PCa

Microbe	Possible mechanism of action
*Cutibacterium acnes*	Induce secretion of cytokines and chemokines such as IL-6 and IL-8 [70, 71], activate transcription factors such as NF-κB and STAT3 [70], and induce production of reactive oxygen species [75], all leading to chronic inflammation and pre-cancerous transformation of the prostatic epithelia.
*E.coli*	Chronic inflammation and tissue damage mediated by CNF1 [81].
*Faecalibacterium prausnitzii*	Down-regulation of pro-inflammatory cytokines TNF-α, TNF-β and IL-6 [82], and increased production of anti-inflammatory cytokine IL-10 [83] in normal tissue.
*Ruminococcus gnavus*	Convert androgen precursors to active androgen enabling alternative source of androgens and resulting in treatment resistance and disease progression [84].

Many studies have also investigated the role of *E. coli* in prostate carcinogenesis. Uro-pathogenic strains of *E. coli* are known to induce prostate tissue damage in rat models of prostatitis [81], mediated by cytotoxic necrotizing factor 1 (CNF1), a virulence factor that has also been shown to promote PCa progression [85]. In a mouse model [52], all mice experimentally infected with *E. coli* for 12 weeks developed chronic inflammation in the prostate, and with prolonged infection showed cytological changes typical for prostatic intraepithelial neoplasia and high-grade dysplasia. Increased epithelial cell proliferation, and oxidative DNA damage was observed in the prostate glands exhibiting dysplasia, together with decreased androgen receptor and *PTEN* gene expression, as compared to the control glands [52]. This could indicate a mechanistic link between *E. coli*-induced inflammation and the onset of PCa or pre-neoplastic lesions.

Others such as *Faecalibacterium prausnitzii* have also been associated with PCa, with higher fecal abundances of *F. prausnitzii* observed in benign as compared to malignant patient samples [59]. *F. prausnitzii* is generally considered to have anti-inflammatory properties with its ability to produce butyrate and induce secretion of anti-inflammatory cytokines such as IL-10, TGF-β2 and IL-1Ra (reviewed by [83]). Furthermore, it has been reported that *F. prausnitzii* also down-regulated the expression of pro-inflammatory cytokines such as TNF-α, TNF-β and IL-6 in lung cancer cell line [82] and could inhibit the phosphorylation of JAK2/STAT3 in breast cancer cells, potentially leading to growth inhibition of cancer cells [86].

### Alterations to host metabolism and immunity mediated by the microbiota

While mechanistic studies investigating microbial regulation of host metabolism in men with PCa are limited, a recent study [84] using PCa mouse models demonstrated that certain species of the intestinal microbiota can modulate the host hormone (e.g. androgen) metabolism and in turn promote cancer growth. Conversely, the study also found that circulating host androgens can alter the composition of the gut microbiota [84]. Notably, the same study showed that *Ruminococcus gnavus* and *Bacteroides acidifaciens* were enriched in the fecal microbiota of castrated (as compared to non-castrated) male mice, and that these species can metabolise androgen precursors, pregnenolone and hydroxypregnenolone, into downstream metabolites of the androgen biosynthesis pathway, dehydroepiandrosterone and testosterone. As prostate tumors are reliant on androgens for continued growth, such an alternate source of androgen could lead to endocrine resistance in PCa patients undergoing castration treatment as shown in PCa mouse models [84].

Disentangling host immune responses against tumor cells from those induced by microorganisms is often difficult. We have recently shown enrichment of several immune cell types within malignant prostate tissue samples having low vs. high abundances of *V. parahaemolyticus* indicating altered host immunity associated with the presence of *V. parahaemolyticus* (Salachan et al., 2022; in press). Moreover, malignant prostate tissue samples having high (vs. low) abundance of *Shewanella* showed decreased enrichment of dendritic cells and down-regulation of several toll-like receptors crucial for an active immune system (Salachan et al., 2022; in press), perhaps enabling tumor-immune evasion. Similarly, another recent study [50] found strong correlation between microbe (e.g. *Delftia acidovorans*, *Gardnerella vaginalis*) abundance in PCa tissue and regulatory T-cells, as well as with down-regulated immune-associated genes including LPCAT2, TL3, and TGFB2, indicating an immuno-suppressive tumor environment associated with the PCa microbiota.

### Future directions

Future research should focus on mapping host microbial species to their functional profiles and delineating specific mechanisms by which microbes enable cancer development and progression or affect treatment response. A better understanding of host-microbiome cross-talk and the associated molecular mechanisms could pave the way for development of novel prevention and/or treatment strategies. Thus, future studies should investigate the clinical utility of targeting the microbiome as a novel mode of anti-cancer treatment. As an example, the genera *Ruminococcus* could be a top candidate for further investigations of its possible driver role in development of treatment resistant CRPC. Conceivably, future prospective clinical trials could test if a novel treatment strategy aimed at eliminating *Ruminococcus sp.* from the gut of men with PCa, is able to prevent/delay treatment resistance and hence improve survival. Future research should also combine metagenomic or metatranscriptomic investigations with metabolomics to provide a more complete overview of host-microbe interactions within the context of PCa. Research should also focus on delineating novel blood and/or urine based microbial signatures that could be of diagnostic, prognostic and/or predictive potential.

## Conclusions

Through this review we aimed to explore the extent of microbial dysbiosis that is associated with PCa by providing an overview of the current knowledge in the field. Moving away from the notion of cancer as solely being a disease of the genome, we believe a more holistic approach towards cancer treatment, informed by genetic, epigenetic, and host-microbiome interactions could benefit treatment decisions in the future. PCa, like other cancers, is a dynamic and heterogeneous disease that has several layers of molecular and cellular complexity associated with it. Microbiome analyses have revealed a bacteria rich environment in the prostate that might be altered during disease onset, progression or treatment. Several species of the microbial community have been associated with PCa aggressiveness and response to therapy, a finding that has also been observed in many other cancer types. A better understanding of the role microbes play in these processes will help us develop novel treatment strategies as well as better risk stratification tools. For instance, it is possible that removal of certain gut microbial species prior to androgen deprivation/hormonal therapy could delay disease progression to CRPC. Several clinical trials are underway looking at e.g. the efficacy of fecal microbiome transplant in combination with established treatment strategies as a means to control tumor progression in different cancers, including in PCa. If successful, these could give us an upper hand in the battle against cancer.

## Data Availability

Not applicable

## Abbreviations

ADT: Androgen deprivation therapy
AN: Adjacent normal
ASV: Amplicon sequence variants
BPH: Benign prostatic hyperplasia
CNF1: Cytotoxic necrotizing factor 1
CRPC: Castration resistant prostate cancer
DGGE: Denaturing gradient gel electrophoresis
EPS: Expressed prostatic secretions
GI: Gastrointestinal
NGS: Next generation sequencing
OTU: Operational taxonomic unit
PCa: Prostate cancer
PRAD: Prostate adenocarcinoma
PSA: Prostate-specific antigen
QC: Quality control
TCGA: The Cancer Genome Atlas
TNM: Tumor-Node-Metastasis
